# Two new species and new records of the ground beetle subgenus Falcinebria Ledoux & Roux, 2005 (Coleoptera, Carabidae, *Nebria*) from Tohoku District, northern Honshu, Japan

**DOI:** 10.3897/BDJ.14.e186108

**Published:** 2026-04-29

**Authors:** Kôji Sasakawa

**Affiliations:** 1 Laboratory of Zoology, Department of Science Education, Faculty of Education, Chiba University, Chiba, Japan Laboratory of Zoology, Department of Science Education, Faculty of Education, Chiba University Chiba Japan

**Keywords:** biogeography, endophallus, male genitalia, morphology, *Nebria hayachinensis*, *Nebria yuda*, new distribution record, taxonomy

## Abstract

**Background:**

Falcinebria Ledoux & Roux, 2005 is a subgenus of Nebria Latreille, 1802 that shows marked differentiation in Japan, but its diversity has not yet been fully elucidated.

**New information:**

This study describes two new species of this subgenus from the Tohoku District in northern Honshu: *N. hayachinensis* sp. nov. from Mt. Hayachine-san, Iwate; and *N. yuda* sp. nov. from Hinosawa, Nishiwaga-machi, Iwate (type locality) and Mt. Mahiru-dake, Akita. Additionally, new records are reported for *N. niohozana* Bates, 1883 and *N. sagittata* Sasakawa, 2020; the records for the former species include its northernmost collection record, while the record for the latter represents its southernmost collection record.

## Introduction

**Figure 1.**
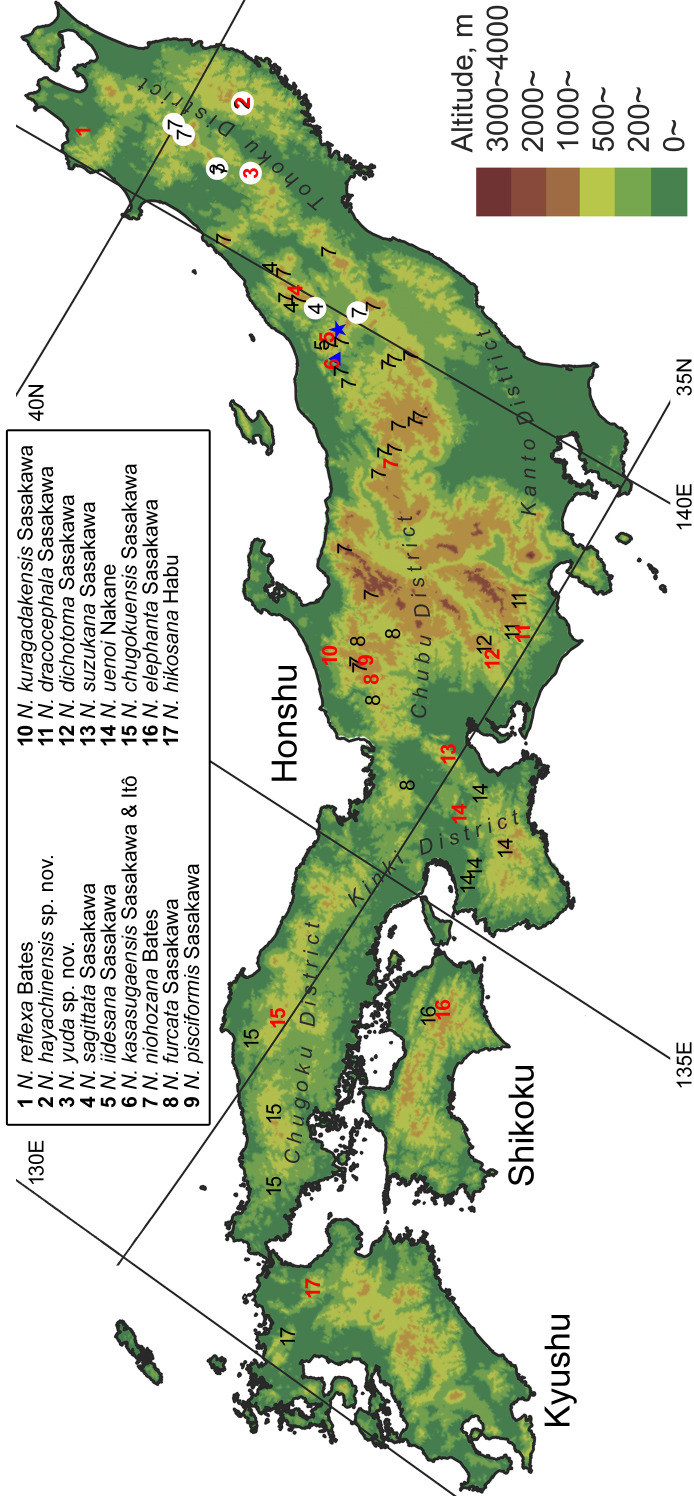
Distribution of species previously treated as *Nebria reflexa* on Honshu, Shikoku and Kyushu, compiled from Sasakawa (2025) and collection sites of specimens examined in the present study (white circles). Sites that are so close to one another as to be difficult to distinguish on the map are combined into a single point. Only records with unambiguous species identity (i.e. collection sites of type materials and records based on specimens identified by the endophallus) are presented. Red letters denote the type localities of each species. The blue star indicates the locality where the sympatric occurrence of *N. iidesana* and *N. niohozana* was confirmed and the blue triangle indicates the locality where the sympatric occurrence of *N. kasasugaensis* and *N. niohozana* was confirmed.

Falcinebria Ledoux & Roux, 2005 is a subgenus o the genus Nebria Latreille, 1802, endemic to East Asia (Huber 2017). This subgenus shows marked regional differentiation due to poor dispersal ability resulting from atrophied hind wings. In Japan, 16 species are known from Honshu, Shikoku and Kyushu (Sasakawa 2023a, Sasakawa 2025). Most of these species are externally very similar and have only recently been distinguished as new species, based on the shape of the male endophallus, which had not been previously examined in this group (Sasakawa 2020, Sasakawa and Itô 2021, Sasakawa 2023a, Sasakawa 2023b, Sasakawa 2025). There are, however, many localities from which no specimens were examined (Fig. 1) and this issue needs to be addressed (Sasakawa 2023a, Sasakawa 2025). The present paper describes two new species of this subgenus from the Tohoku District in northern Honshu, where specimens of this group have hitherto been insufficiently examined. Additionally, new distributional records of two known species, *N. niohozana* Bates, 1883 and *N. sagittata* Sasakawa, 2020, are reported.

## Materials and methods

Information on locally adjacent related species (*N. reflexa*, *N. sagittata* and *N. iidesana* Sasakawa, 2020) was obtained from Sasakawa (2020), who described key morphological characters, based on materials including type specimens and from scaled photographs taken in his study. Body length (BL) was measured from mandible apex to elytral end.

For the new species, all male specimens were dissected to examine the structure of the endophallus of the genitalia. The utility of this male genital morphology for species-level taxonomy has been demonstrated in many taxa within Carabidae (e.g. Janovska et al. (2013)). The endophallus was everted by injecting toothpaste (White & White; LION, Tokyo, Japan) from the base of the aedeagus using an insulin syringe fitted with a pre-attached 29-gauge needle (SS-10M2913; TERUMO, Tokyo, Japan).

For the endophallus of species other than *N. hayachinensis* sp. nov. and *N. reflexa*, each set of photographs was taken from the following four angles, from left to right: right lateral view, ventral view, dorsal view and posterodorsal view (to show the dorsal aspect of the dorsoapical lobe). For *N. hayachinensis* and *N. reflexa*, in addition to photographs taken from these four angles, a left dorsolateral view of the dorsoapical lobe was also taken. Terminology of endophallus structures followed Sasakawa (2020).

The following abbreviations are used for the male endophallus structures: da = dorsoapical lobe; db = dorsobasal lobe; dm = dorsomedian lobe; go = gonopore; gp = gonopore protrusion; la = left lateroapical lobe; lb = left laterobasal lobe; ra = right lateroapical lobe; rb = right laterobasal lobe; vb = ventrobasal swelling. Endophallus structures that were not fully everted are indicated by an asterisk (*).

Holotypes of the new species are deposited in the collections of Iwate Prefectural Museum, Morioka, Iwate, Japan (IPM). Other specimens are deposited in the collections of Kitakyushu Museum of Natural History & Human History, Fukuoka, Japan (KMNH), Museum of Nature and Human Activities, Hyogo, Japan (MNHAH), Yonezawa Insectarium, Yonezawa-shi, Yamagata, Japan (YONE) and IPM.

## Taxon treatments

### Nebria (Falcinebria) hayachinensis sp. nov.

919560F9-B8B5-5082-8731-7AEE0B326DF3

E1121A9E-1069-4B7D-9DE6-989B8AFCEA4D

#### Materials

- **Type status:** Holotype. **Occurrence:** catalogNumber: 111294; recordedBy: T. Oku; individualCount: 1; sex: male; lifeStage: adult; occurrenceID: 4A58BF70-4078-5112-A02F-59AC5D4BA47E; **Taxon:** kingdom: Animalia; phylum: Arthropoda; class: Insecta; order: Coleoptera; family: Carabidae; genus: Nebria; subgenus: Falcinebira; specificEpithet: hayachinensis; **Location:** country: Japan; stateProvince: Iwate; locality: Mt. Hayachine-san, near Odagoe; verbatimLocality: Water source near the entrance to the Odagoe Trail, HONSHU, Mt. Hayachine, Iwate Pref.; **Event:** samplingProtocol: none specified; eventDate: 27-07-1978; **Record Level:** institutionCode: IPM
- **Type status:** Paratype. **Occurrence:** catalogNumber: 111293; recordedBy: T. Oku; individualCount: 1; sex: male; lifeStage: adult; occurrenceID: D62F9DA6-0AE9-5DA6-A187-48667D820B26; **Taxon:** kingdom: Animalia; phylum: Arthropoda; class: Insecta; order: Coleoptera; family: Carabidae; genus: Nebria; subgenus: Falcinebira; specificEpithet: hayachinensis; **Location:** country: Japan; stateProvince: Iwate; municipality: Hanamaki-shi; locality: Mt. Hayachine-san, Kawara-no-bô; verbatimLocality: Kawarano-bō, Mt. Hayachine, Iwate, Honshu; **Event:** samplingProtocol: none specified; eventDate: 05-06-1977; **Record Level:** institutionCode: IPM

#### Description

**External structures** (Fig. 2a). BL 8.98 mm for holotype male and 9.02 mm for paratype male. Other structures as in related species previously regarded as *Nebria reflexa* (Sasakawa 2020, Sasakawa 2025). **Male genitalia** (Fig. 3a). Ventral surface of aedeagal apex not concave. Dorsomedian lobe semi-prolate-spheroid; apex directed dorsally. Dorsoapical lobe with semi-prolate-spheroid protrusion (a_1_) at basal part, which is slightly bent towards endophallus base; dorsomedian area with conical-shaped protrusion (a_2_), apex of which is widely rounded and directed right-dorsolaterally; apical portion (a_3_) of stout cylindrical form, with apex abruptly narrowed and directed left-laterally. Right and left laterobasal lobes semi-prolate-spheroid. Right lateroapical lobe almost semi-spherical; apex directed right-laterally. Left lateroapical lobe bifurcated at base; apical branch large and directed left-laterally; basal branch similar in size to right laterobasal lobe and adjoining it. Ventrobasal area widely swollen. Relative sizes of lobe and protrusions are as follows: protrusion on dorsomedian area of dorsoapical lobe (a_1_) ≤ protrusion on basal part of dorsoapical lobe (a_2_) ≈ left laterobasal lobe ≈ basal branch of left lateroapical lobe ≈ dorsomedian lobe < right lateroapical lobe ≈ right laterobasal lobe < apical branch of left lateroapical lobe < apical portion of dorsoapical lobe (a_3_).

#### Diagnosis

Similar to the locally adjacent species *N. reflexa* (Sasakawa 2020) and *N. yuda* sp. nov. (Fig. 2b), but distinguished from the former by larger dorsoapical lobe of endophallus (Fig. 3) and from the latter by smaller body size.

#### Etymology

The specific name is derived from Mt. Hayachine-san, the type locality of the new species.

#### Notes

Amongst the known related species, this new species is considered to be most closely related to the locally adjacent species *N. reflexa*, as the two species share basic structures of the endophallus, with homology readily traceable (Fig. 3). These two species also share the trait of having a smaller body size compared with other closely-related species (Sasakawa 2020, this study).

### Nebria (Falcinebria) yuda sp. nov.

07B164B7-A84E-5FFA-AFFD-6FFA48AB2AE1

43A1CD71-2E4E-4C3A-9956-032E19FC098B

#### Materials

- **Type status:** Holotype. **Occurrence:** catalogNumber: 111291; recordedBy: M. Shōji; individualCount: 1; sex: male; lifeStage: adult; occurrenceID: 142E2B85-A2A1-59E1-9553-3D42037B2772; **Taxon:** kingdom: Animalia; phylum: Arthropoda; class: Insecta; order: Coleoptera; family: Carabidae; genus: Nebria; subgenus: Falcinebira; specificEpithet: yuda; **Location:** country: Japan; stateProvince: Iwate; municipality: Nishiwaga-machi; locality: Hinosawa; verbatimLocality: Washiai mori, Yuda; **Event:** samplingProtocol: none specified; eventDate: 03-08-1966; **Record Level:** institutionCode: IPM
- **Type status:** Paratype. **Occurrence:** catalogNumber: KMNH_KS_017; recordedBy: M. Fujioka; individualCount: 1; sex: male; lifeStage: adult; occurrenceID: 5FC3989A-91ED-5DC7-937E-ABA046094FC2; **Taxon:** kingdom: Animalia; phylum: Arthropoda; class: Insecta; order: Coleoptera; family: Carabidae; genus: Nebria; subgenus: Falcinebira; specificEpithet: yuda; **Location:** country: Japan; stateProvince: Akita; municipality: Misaso-chô; locality: Naniwa, Mt. Mahiru-dake, Mahirudake-rindô; verbatimLocality: Mariru-rindô, Senboku, Akita Pref.; locationRemarks: "Mariru" on the original label is probably a misspelling of "Mahiru."; **Event:** samplingProtocol: none specified; eventDate: 30-07-1985; **Record Level:** institutionCode: KMNH
- **Type status:** Paratype. **Occurrence:** catalogNumber: KMNH_KS_018; recordedBy: M. Fujioka; individualCount: 1; sex: female; lifeStage: adult; occurrenceID: 2BD70B53-A4CC-5CDA-A9CD-B35CC52FB8CB; **Taxon:** kingdom: Animalia; phylum: Arthropoda; class: Insecta; order: Coleoptera; family: Carabidae; genus: Nebria; subgenus: Falcinebira; specificEpithet: yuda; **Location:** country: Japan; stateProvince: Akita; municipality: Misaso-chô; locality: Naniwa, Mt. Mahiru-dake, Mahirudake-rindô; verbatimLocality: Mahiru-rindô, Senboku, Akita Pref.; **Event:** samplingProtocol: none specified; eventDate: 30-07-1985; **Record Level:** institutionCode: KMNH

#### Description

**External structures** (Fig. 2b). BL 9.54 mm for holotype male, 9.33 mm for paratype male and 9.39 mm for paratype female. Other structures as in related species, previously regarded as *Nebria reflexa* (Sasakawa 2020, Sasakawa 2025). **Male genitalia** (Fig. 4a). Ventral surface of aedeagal apex not concave. Dorsobasal lobe small, protruding. Dorsomedian lobe with apex truncate in lateral view. Dorsoapical lobe with basal part protruding and weakly bent towards endophallus base; apical portion bifid in dorsal view, with left branch truncate at apex and wider than right branch, which is simple in shape and not truncate at apex. Right and left laterobasal lobes semi-prolate-spheroid. Right lateroapical lobe conical with wide base; subapical part weakly constricted. Left lateroapical lobe bifurcated at base; apical branch larger and directed left-laterally; basal branch smaller, similar in size to right laterobasal lobe and adjoining it. Ventrobasal area widely swollen. Gonopore protrusion cylindrical, weakly bent at middle towards endophallus apex. Relative sizes of lobe and protrusions are as follows: dorsobasal lobe ≤ basal branch of left lateroapical lobe ≤ right laterobasal lobe ≈ left laterobasal lobe ≈ apical branch of left lateroapical lobe < dorsomedian lobe ≈ right lateroapical lobe ≤ protrusion on basal part of dorsoapical lobe < gonopore protrusion ≈ apical portion of dorsoapical lobe.

#### Diagnosis

Similar to the locally adjacent species *N. reflexa* (Sasakawa 2020), *N. hayachinensis* (Fig. 2a) and *N. sagittata* (Sasakawa 2020), but distinguished from *N. reflexa* and *N. hayachinensis* by larger body size and from *N. sagittata* by bifurcated left lateroapical lobe of endophallus (Fig. 4).

#### Etymology

The specific name is derived from “Yuda,” a former town name to which the area including the type locality belonged.

#### Notes

Amongst the endophallus structures of this new species, the bifurcated left lateroapical lobe is similar to those of *N. reflexa* and *N. hayachinensis*, which are considered sister taxa (this study). In contrast, the arrow-shaped dorsoapical lobe is similar to those of *N. sagittata* (Fig. 4) and *N. iidesana* (Sasakawa 2020), which are also considered sister taxa (Sasakawa 2020, Sasakawa and Itô 2021). Thus, the new species exhibits a mosaic combination of characters in the endophallus from the *N. reflexa–N. hayachinensis* clade and the *N. sagittata–N. iidesana* clade and is inferred to occupy a phylogenetic position linking these two clades. This interpretation is consistent with the fact that the distribution of the new species lies between those of the two clades (Fig. 1).

### Nebria (Falcinebria) sagittata

Sasakawa, 2020

BF22856B-7EAF-5CED-9CBB-494883F2B1C3

#### Materials

- **Type status:** Other material. **Occurrence:** catalogNumber: cop 12465; recordedBy: K. Kusakari; individualCount: 1; sex: male; lifeStage: adult; occurrenceID: 8C4F592C-186F-5594-A611-8504F1836D42; **Taxon:** kingdom: Animalia; phylum: Arthropoda; class: Insecta; order: Coleoptera; family: Carabidae; genus: Nebria; subgenus: Falcinebira; specificEpithet: sagittata; **Location:** country: Japan; stateProvince: Yamagata; municipality: Oguni-machi; locality: Kaname, Kanamegawa River; verbatimLocality: Upper reaches of Kanamegawa River, Oguni-machi; **Event:** samplingProtocol: none specified; eventDate: 05-08-1993; **Record Level:** institutionCode: YONE

#### Notes

The previous southernmost collection record of this species was “Asahi-mura,” a part of Murakami-shi, Niigata Prefecture (Sasakawa 2020). The present collection site is located south of “Asahi-mura” and, therefore, represents the new southernmost collection record for the species. No distinct differences in the endophallus structure were observed between specimens from the southernmost collection site and other localities (Fig. 4b; see also Sasakawa (2020)).

### Nebria (Falcinebira) niohozana

Bates, 1883

8F951D1A-3D15-5B4C-86F8-131DB72E9DA6

#### Materials

- **Type status:** Other material. **Occurrence:** catalogNumber: B1-160748~160749; recordedBy: M. Fujioka; individualCount: 2; sex: male; lifeStage: adult; occurrenceID: F3998A49-F112-57E9-B4D4-1C887C9FB78E; **Taxon:** kingdom: Animalia; phylum: Arthropoda; class: Insecta; order: Coleoptera; family: Carabidae; genus: Nebria; subgenus: Falcinebira; specificEpithet: niohozana; **Location:** country: Japan; stateProvince: Akita; municipality: Kazuno-shi; locality: Mt. Hchimantai; verbatimElevation: 1500 m; **Event:** samplingProtocol: none specified; eventDate: 20-09-1986; **Record Level:** institutionCode: MNHAH
- **Type status:** Other material. **Occurrence:** catalogNumber: 111295(1)~(10), (15), (17); recordedBy: K. Satake; individualCount: 11; sex: 9 males, 2 females; lifeStage: adult; occurrenceID: 66CC9506-8330-5435-AD9F-95D8F109F82B; **Taxon:** kingdom: Animalia; phylum: Arthropoda; class: Insecta; order: Coleoptera; family: Carabidae; genus: Nebria; subgenus: Falcinebira; specificEpithet: niohozana; **Location:** country: Japan; stateProvince: Iwate; locality: Mt. Hchimantai; verbatimLocality: Hachimantai, 1500 m, Iwate Pref.; verbatimElevation: 1500 m; **Event:** samplingProtocol: none specified; eventDate: 07-07-1987; **Record Level:** institutionCode: IPM
- **Type status:** Other material. **Occurrence:** catalogNumber: 111295(3), (11), (12), (16); recordedBy: K. Satake; individualCount: 4; sex: 1 male, 3 females; lifeStage: adult; occurrenceID: C31D96DD-D0E2-5323-8615-F34FA1612E1E; **Taxon:** kingdom: Animalia; phylum: Arthropoda; class: Insecta; order: Coleoptera; family: Carabidae; genus: Nebria; subgenus: Falcinebira; specificEpithet: niohozana; **Location:** country: Japan; stateProvince: Iwate; locality: Mt. Hchimantai; verbatimLocality: Hachimantai, 1500 m, Iwate Pref.; verbatimElevation: 1500 m; **Event:** samplingProtocol: none specified; eventDate: 17-07-1987; **Record Level:** institutionCode: IPM
- **Type status:** Other material. **Occurrence:** catalogNumber: 111292(1)~(3); recordedBy: T. Chiba; individualCount: 3; sex: males; lifeStage: adult; occurrenceID: D9249EF2-2062-5D74-A799-B3CC27FA4FB8; **Taxon:** kingdom: Animalia; phylum: Arthropoda; class: Insecta; order: Coleoptera; family: Carabidae; genus: Nebria; subgenus: Falcinebira; specificEpithet: niohozana; **Location:** country: Japan; stateProvince: Iwate; municipality: Shizukuishi-cho; locality: Nishine, Takinoue Spa; verbatimLocality: TaKiNoUE; **Event:** samplingProtocol: none specified; eventDate: 24-06-1967; **Record Level:** institutionCode: IPM
- **Type status:** Other material. **Occurrence:** catalogNumber: cop 12467; recordedBy: B. Yamaya; individualCount: 1; sex: female; lifeStage: adult; occurrenceID: 397670EF-F1E8-5E29-B507-E4F68503078F; **Taxon:** kingdom: Animalia; phylum: Arthropoda; class: Insecta; order: Coleoptera; family: Carabidae; genus: Nebria; subgenus: Falcinebira; specificEpithet: niohozana; **Location:** country: Japan; stateProvince: Yamagata; locality: Mt. Chokai-san; verbatimLocality: Mt. Tyokai Yamagata-pref.; **Event:** samplingProtocol: none specified; eventDate: 01-09-1979; **Record Level:** institutionCode: YONE
- **Type status:** Other material. **Occurrence:** catalogNumber: cop 12466; recordedBy: B. Yamaya; individualCount: 1; sex: male; lifeStage: adult; occurrenceID: 65B7D088-7D75-5958-B0D4-86B9FB36061E; **Taxon:** kingdom: Animalia; phylum: Arthropoda; class: Insecta; order: Coleoptera; family: Carabidae; genus: Nebria; subgenus: Falcinebira; specificEpithet: niohozana; **Location:** country: Japan; stateProvince: Yamagata; locality: Mt. Gassan; verbatimLocality: Gathu-san Yamagata-pref; **Event:** samplingProtocol: none specified; eventDate: 19-09-1982; **Record Level:** institutionCode: YONE
- **Type status:** Other material. **Occurrence:** catalogNumber: cop 12459~12461; recordedBy: K. Kusakari; individualCount: 3; sex: 1 male, 2 females; lifeStage: adult; occurrenceID: 6890B6CB-FB0A-5EB5-B99C-C1A98789BC3C; **Taxon:** kingdom: Animalia; phylum: Arthropoda; class: Insecta; order: Coleoptera; family: Carabidae; genus: Nebria; subgenus: Falcinebira; specificEpithet: niohozana; **Location:** country: Japan; stateProvince: Yamagata; municipality: Yonezawa-shi; locality: Seki, Shirabu Spa; verbatimLocality: Sirabu-Takayu Yonezawa-Si; **Event:** samplingProtocol: none specified; eventDate: 26-06-1994; **Record Level:** institutionCode: YONE
- **Type status:** Other material. **Occurrence:** catalogNumber: cop 12455, 12456; recordedBy: K. Kusakari; individualCount: 2; sex: males; lifeStage: adult; occurrenceID: 424BB1FF-CA6D-5777-97A8-C06C074F76BF; **Taxon:** kingdom: Animalia; phylum: Arthropoda; class: Insecta; order: Coleoptera; family: Carabidae; genus: Nebria; subgenus: Falcinebira; specificEpithet: niohozana; **Location:** country: Japan; stateProvince: Yamagata~Fukushima; locality: Babayachi~Mt. Higashidaiten; verbatimLocality: Babayachi~Higashidaiten; **Event:** samplingProtocol: none specified; eventDate: 23-06-1991; **Record Level:** institutionCode: YONE
- **Type status:** Other material. **Occurrence:** catalogNumber: cop 12463, 12464; recordedBy: K. Kusakari; individualCount: 2; sex: males; lifeStage: adult; occurrenceID: 31563785-ACBE-5A45-8CFD-17F8221B7CB0; **Taxon:** kingdom: Animalia; phylum: Arthropoda; class: Insecta; order: Coleoptera; family: Carabidae; genus: Nebria; subgenus: Falcinebira; specificEpithet: niohozana; **Location:** country: Japan; stateProvince: Yamagata~Fukushima; locality: Ningyosaki~Mt. Nishidaiten; verbatimLocality: Ningyosaki~Nishidaiten; **Event:** samplingProtocol: none specified; eventDate: 14-08-1992; **Record Level:** institutionCode: YONE

#### Notes

The previous northernmost collection record of this species was Mt. Mahiru-dake (Nakaya 2024). Amongst the collection sites in the present study, Mt. Hachimantai and Takinoue Spa are located north of Mt. Mahiru-dake, expanding the known distribution of the species (Fig. 1). Of these two sites, the record from Mt. Hachimantai represents the northernmost collection record for the species and the first record from Akita Prefecture (Sasakawa 2020). No distinct differences in the endophallus structure were observed between specimens from the northernmost collection site and other localities (Fig. 5; see also Sasakawa 2020, Nakaya 2024, Sasakawa 2025).

## Supplementary Material

XML Treatment for Nebria (Falcinebria) hayachinensis

XML Treatment for Nebria (Falcinebria) yuda

XML Treatment for Nebria (Falcinebria) sagittata

XML Treatment for Nebria (Falcinebira) niohozana

## Figures and Tables

**Figure 2a.**
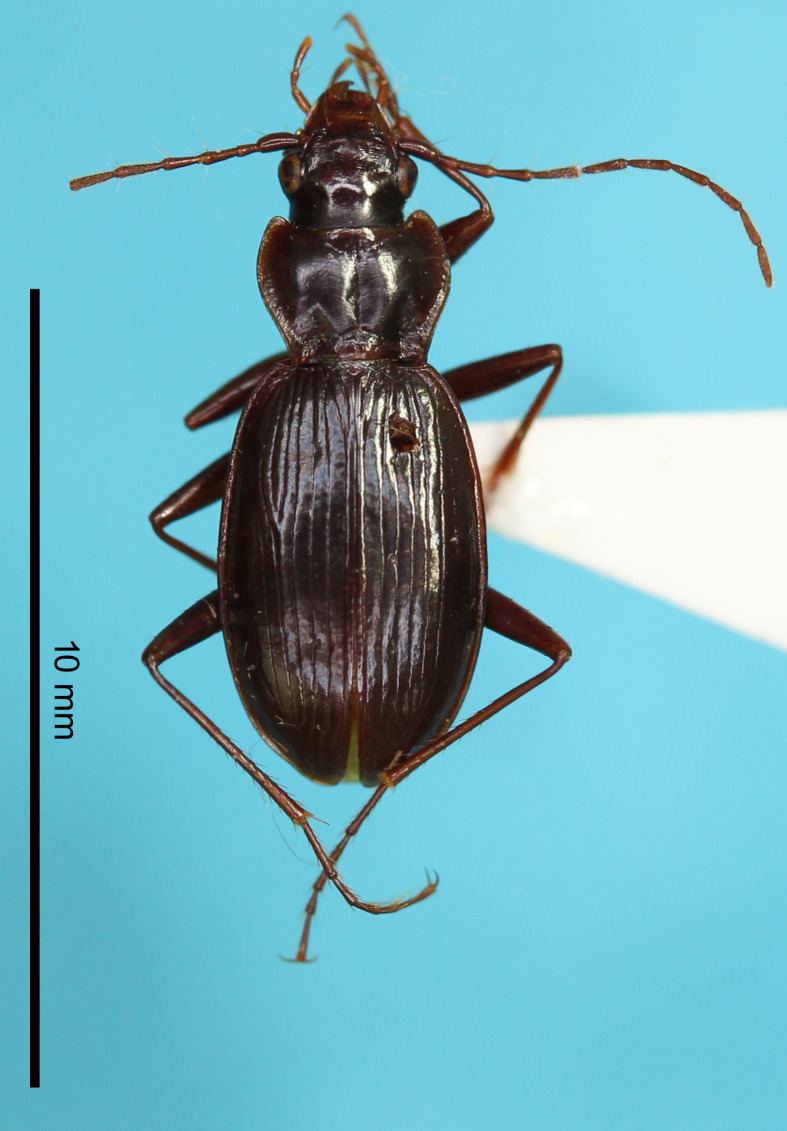
*N. hayachinensis* sp. nov., holotype male;

**Figure 2b.**
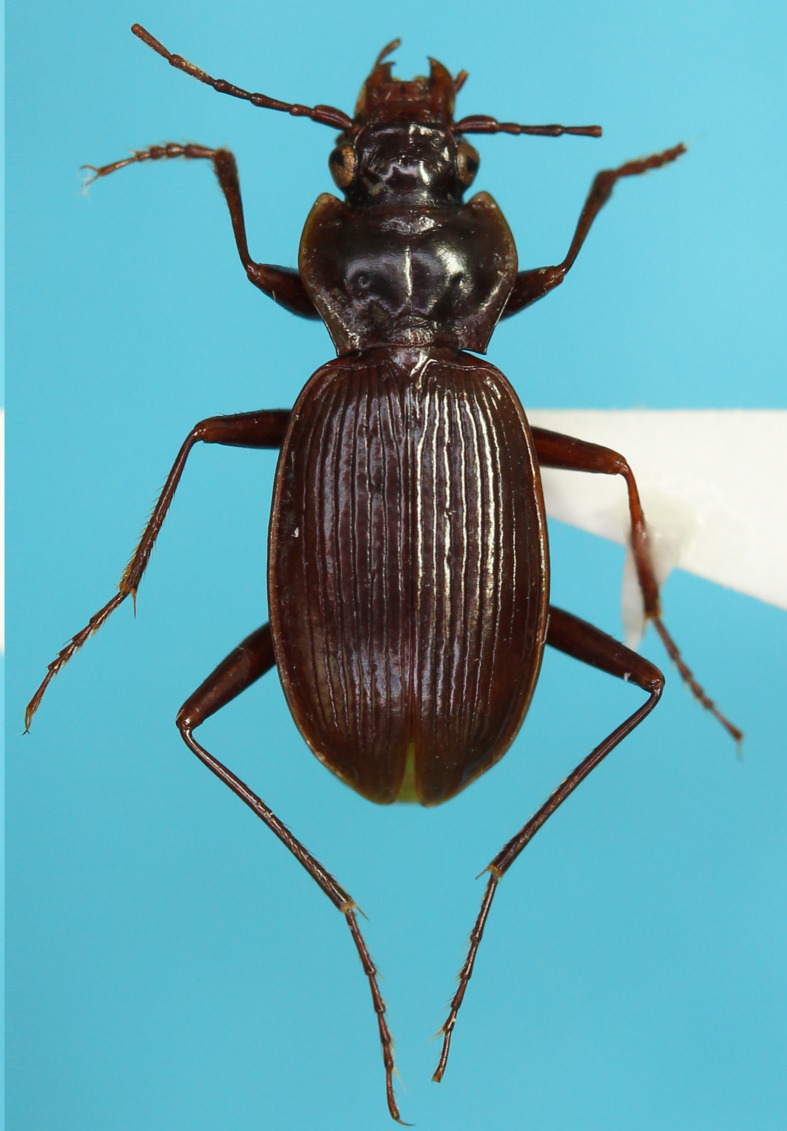
*N. yuda* sp. nov., holotype male;

**Figure 2c.**
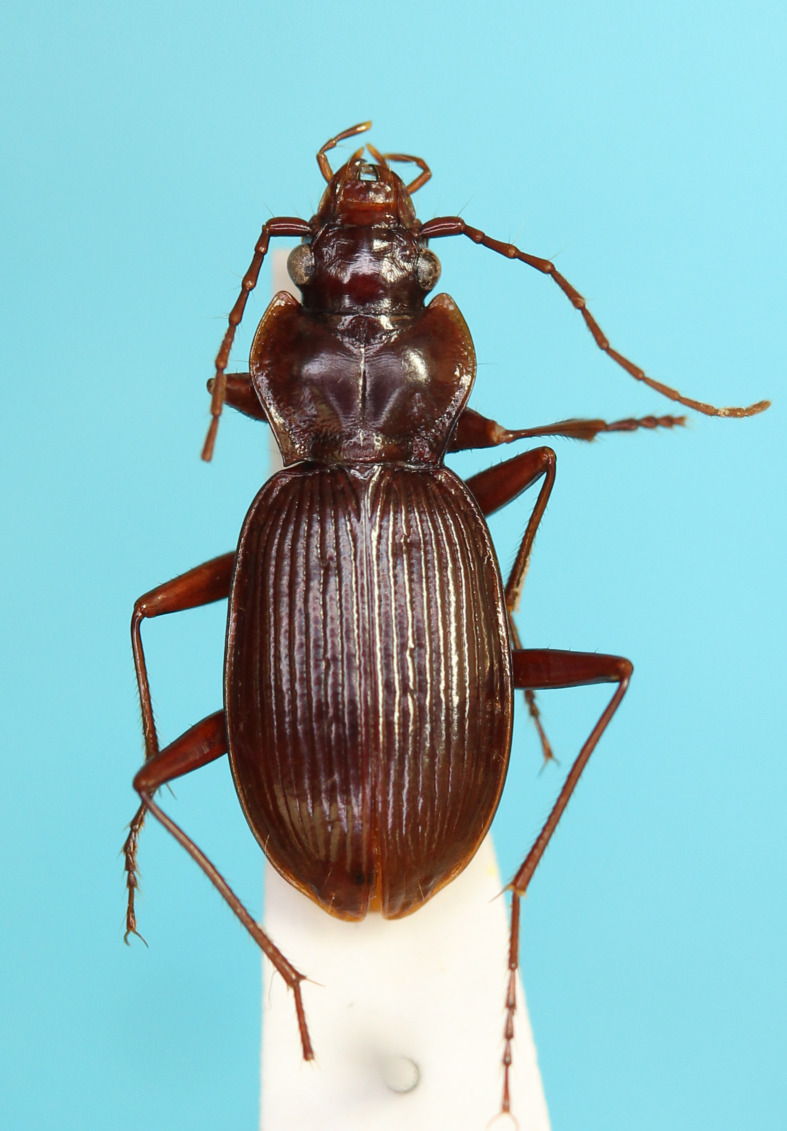
*N. sagittata*, male from Kanamegawa River;

**Figure 2d.**
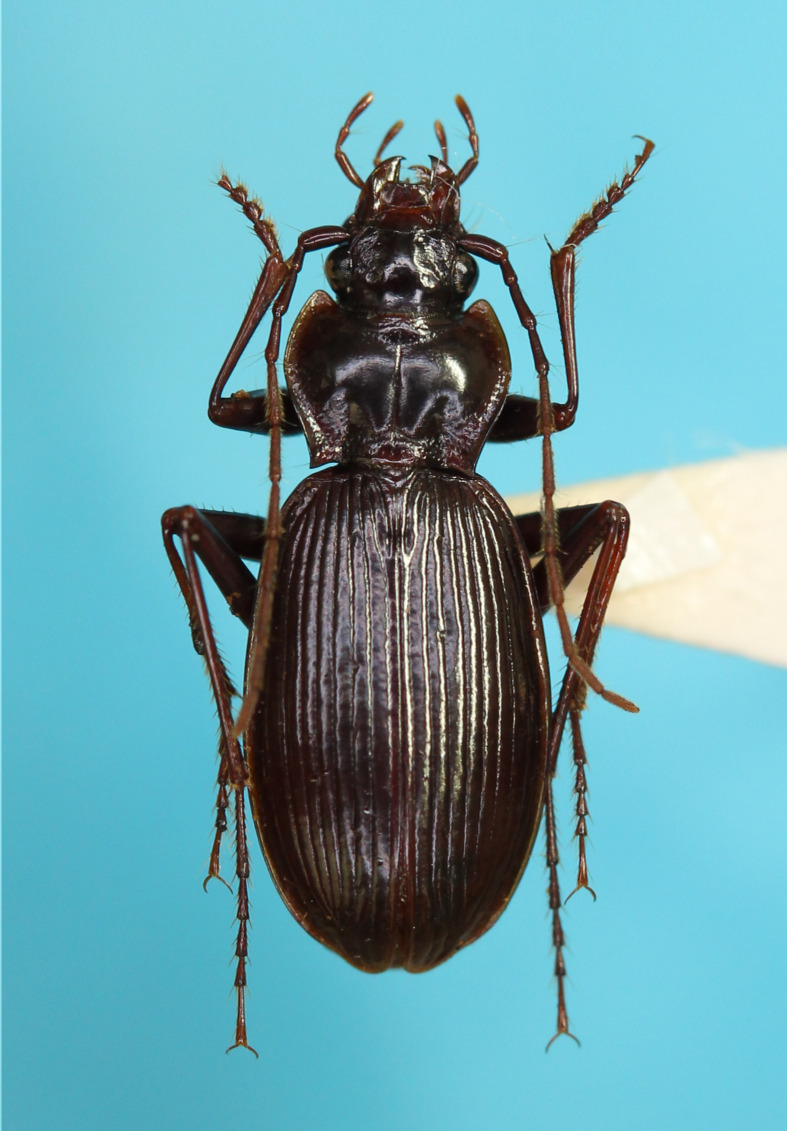
*N. niohozana*, male from Mt. Hachimantai.

**Figure 3a.**
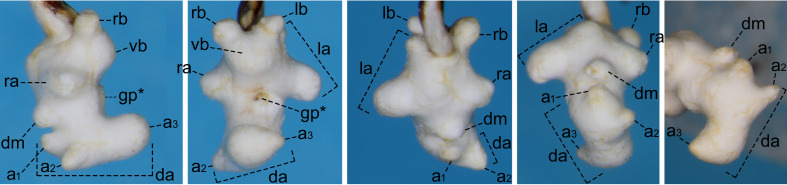
*N. hayachinensis* sp. nov., holotype male;

**Figure 3b.**
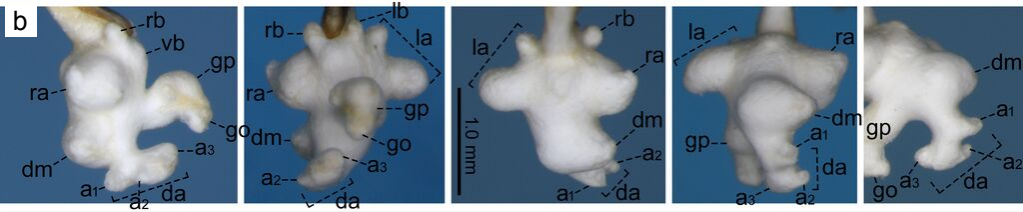
*N. reflexa*, male from type locality.

**Figure 4a.**
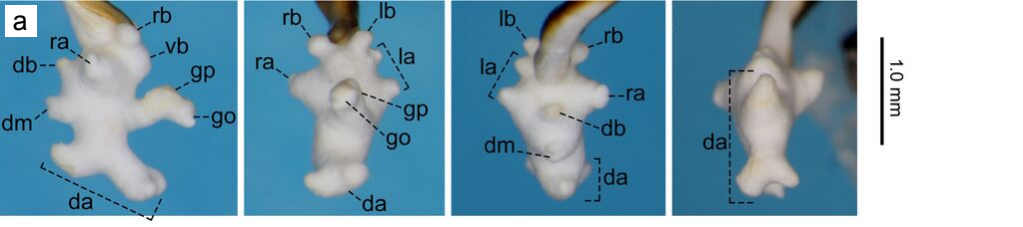
*N. yuda* sp. nov., holotype male;

**Figure 4b.**
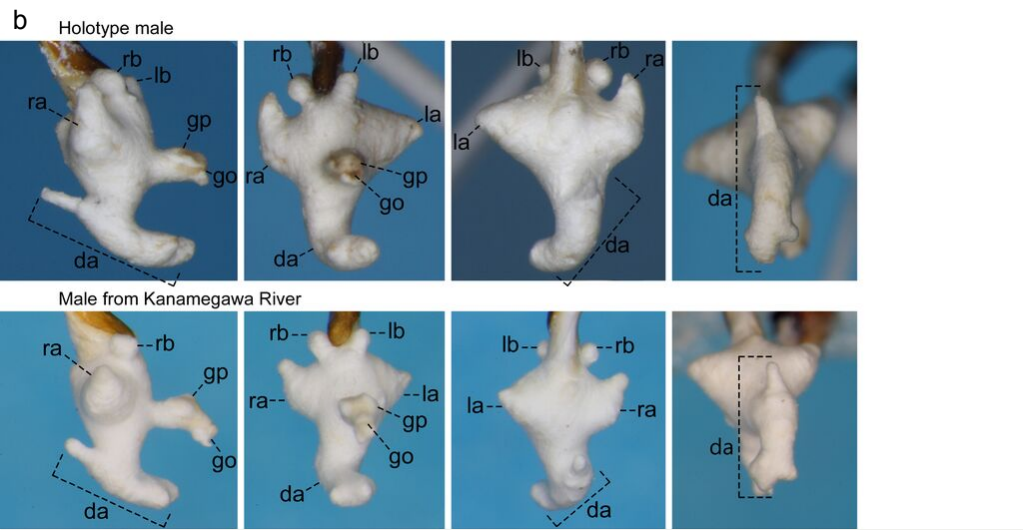
*N. sagittata*, holotype male (upper) and male from Kanamegawa River (lower).

**Figure 5a.**
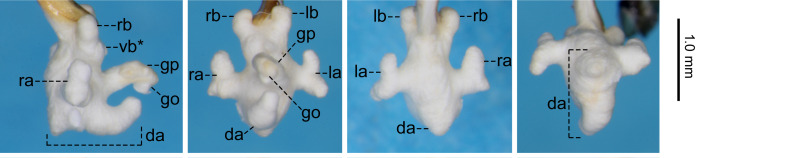
Male from Mt. Hachimantai;

**Figure 5b.**
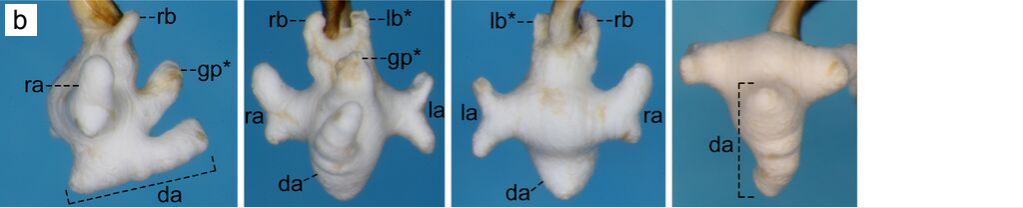
Male from Ningyosaki ~ Mt. Nishidaiten.

## References

[B13826866] Huber C, Löbl I, Löbl D (2017). Catalogue of Palaearctic Coleoptera. Vol. 1. Archostemata – Myxophaga – Adephaga. Revised and Updated Edition.

[B13925462] Janovska М., Anichtchenko А. V., Erwin T. (2013). Significant new taxonomic tool for Carabidae (Insecta: Coleoptera): endophallus inflation methods revised. Caucasian Entomological Bulletin.

[B13846499] Nakaya N. (2024). A new distributional record of Nebria (Falcinebria) niohozana Bates, 1883 (Coleoptera, Carabidae) from Iwate Prefecture, Honshu, Japan. Niche Life.

[B13826821] Sasakawa Kôji (2020). Taxonomic studies of the ground beetle 
subgenus Falcinebria Ledoux & Roux, 2005 (Coleoptera, Carabidae, *Nebria*) from Honshû, Japan. ZooKeys.

[B13826857] Sasakawa Kôji, Itô Hirotarô (2021). A new species and distribution record of the ground beetle 
subgenus Falcinebria Ledoux & Roux, 2005 (Coleoptera: Carabidae: *Nebria*) from central Honshu, Japan.. Biogeography.

[B13826830] Sasakawa Kôji (2023). Taxonomic study of the alpine carabid beetle Nebria (Falcinebria) taketoi Habu, 1962 (Coleoptera, Carabidae). Alpine Entomology.

[B13826839] Sasakawa Kôji (2023). A new and little-known species of the ground beetle genus Nebria
subgenus
Falcinebria Ledoux and Roux, 2005 (Coleoptera: Carabidae) from Japan. Taxonomy.

[B13826848] Sasakawa Kôji (2025). Taxonomic studies of the ground beetle 
subgenus Falcinebria Ledoux & Roux, 2005 (Coleoptera, Carabidae, *Nebria*) from the Japanese Alps (central Honshu), Shikoku, and Kyushu, Japan. ZooKeys.

